# Gestational Diabetes Mellitus and Small-for-Gestational-Age: An Insight into the Placental Molecular Biomarkers

**DOI:** 10.3390/ijms24032240

**Published:** 2023-01-23

**Authors:** Christian Giommi, Marta Lombό, Nina Montik, Michela Paolucci, Valentina Notarstefano, Giovanni Delli Carpini, Andrea Ciavattini, Antonio Ragusa, Francesca Maradonna, Elisabetta Giorgini, Oliana Carnevali

**Affiliations:** 1 Department of Life and Environmental Sciences, Università Politecnica delle Marche, 60131 Ancona, Italy; 2 INBB—Consorzio Interuniversitario di Biosistemi e Biostrutture, 00136 Roma, Italy; 3 Department of Molecular Biology, Faculty of Biology and Environmental Sciences, Universidad de León, 24071 León, Spain; 4 Department of Odontostomatological and Specialized Clinical Sciences, Università Politecnica delle Marche, 60020 Ancona, Italy; 5 Department of Obstetrics and Gynecology, Università Campus Bio Medico di Roma, 00128 Roma, Italy

**Keywords:** FTIRI, placenta, GDM, SGA, endocannabinoid receptors

## Abstract

Gestational diabetes mellitus (GDM) and small-for-gestational-age (SGA) are two metabolic-related diseases that could affect women during pregnancy. Considering that the chorionic villi (CVs) are crucial structures for the feto-maternal exchange, the alterations in their conformation have been linked to an imbalanced metabolic environment of placenta. In this study, a multidisciplinary approach has been carried out to describe the changes occurring in the placental CVs of GDM and SGA patients. The results revealed higher levels of superoxide dismutase 1 (SOD-1) and catalase (CAT), especially in the GDM placentae, which could be correlated with the hyperglycemic environment characteristic of this pathology. Furthermore, spectroscopy and histologic analyses revealed that both pathologies modify the placental lipid composition altering its structure. However, SGA induces lipid peroxidation and reduces collagen deposition within the CVs. Since the endocannabinoid system (ECS) is involved in placentation and different metabolic activities, the cannabinoid receptor 1 (CB1) and transient receptor potential cation channel subfamily V member 1 (TRPV-1) were analyzed. No changes have been observed either at general or specific levels in the CVs comparing control and pathological samples, suggesting the non-involvement of the cannabinoid system in these two pathologies.

## 1. Introduction

Placenta, representing the interface between the mother and the fetus, is a metabolically active organ that allows the transport of a wide range of nutrients and micronutrients, the elimination of catabolites, and the production of hormones, thus sustaining the proper growth of the fetus [1,2]. Therefore, metabolic alterations in this organ have been related to the onset of some pathological conditions, such as gestational diabetes mellitus (GDM) and small-for-gestational-age (SGA).

GDM is one of the most common gestational pathologies, affecting 7–10% of pregnant woman worldwide [3,4]. The consequences derived from GDM for the fetus are a higher risk of macrosomia, icterus, glycemic disorders, and obesity [5,6]. GDM is characterized by hyperglycemia, and its diagnosis could be undertaken at any time during pregnancy by using the oral glucose tolerance test (OGTT), which is based on a glycemia curve built up with blood glucose values collected before and after consumption of a 75 g glucose dose [3,4,7]. The defined reference parameters for the OGTT test are: 92 mg/dL during fasting, 180 mg/dL one hour after drinking the glucose solution, and 153 mg/dL two hours after drinking the glucose solution. Then, GDM is diagnosed in pregnant women having at least one of the above parameters higher than the reference values [4,8]. An increased body mass index (BMI) during early pregnancy is a risk factor for GDM. Still, independently of the BMI, a prolonged excessive caloric intake, overwhelming the capacity of pancreas β-cells to produce insulin, leads to changes in insulin sensitivity [6]. 

On the other hand, SGA is a clinical condition characterized by fetal weight under the 10th percentile considering the age of gestation [9]. Since the diagnosis of SGA relies on the fetus weight and length [10], ultrasounds are commonly used to study the fetus biometry for the identification of fetal growth restriction (FGR), a condition that could result in SGA at delivery [11]. In addition, the curve of fetal growth must be customized for the patients to discriminate between constitutionally normal SGA newborns, which are healthy, and SGA patients due to FGR [11]. The recombinant human growth hormone (rhGH) has been approved in Europe for the treatment of this condition after birth, but the response to this therapy is highly variable [12]. In fact, most SGA infants have normal levels of GH but display lower levels of circulating insulin-like growth factor 1 (IGF-1) concentrations, as a result of *IGF1* mRNA reduction. However, the levels of their binding proteins (IGFBPs) are higher, pointing to a lesser availability for the receptor binding [13]. Furthermore, these changes have been also linked to a hypermethylation of *IGF1* promoter and a decreased CpG methylation of *IGFBPs* promoters, suggesting that epimutations are involved in the pathophysiology of this condition [13]. Moreover, low levels of maternal folate have been related to a higher incidence of SGA since they lead not only to a placental dysfunction but also to a reduction in amino acid transport and alterations in placental hormone levels [14]. This placental dysfunction mainly consists of an impaired perfusion that triggers hypoxia; thus, SGA newborns usually present increased red blood cells, decreased body temperature, and glucose blood levels, having a higher risk of mortality and morbidity during the neonatal period and beyond [10].

Chorionic villi (CV), composed of cytotrophoblast and syncytiotrophoblast, are the metabolic functional unit of placenta [15]. Since both GDM and SGA are caused by changes in metabolic activities, functional and structural alterations in term placental can be evaluated by non-invasive approaches. In this regard, GDM has been associated with morphological modifications in the placenta, histological changes, increased placental intervillous space and terminal villi volume, and fibrinoid areas and glycogen deposition, the last being the most evident [16]. Concerning metabolism, despite no observable metabolic alterations were found in isolated syncytiotrophoblast cells in GDM, cytotrophoblast cells isolated from GDM placentae showed lower glycolysis and ATP content compared to CV samples from normal pregnancy, concomitant with bigger lipid droplets [17]. Due to the above-mentioned alterations, inflammation and oxidative stress are increased in GDM placenta, leading to fetal hypoxia [18]. SGA has been also associated with a reduced placental elongase level and an increased desaturase activity, with consequent differences in fatty acid composition and omega-6/omega-3 ratio increase [19].

Endocannabinoids, endogenous bioactive lipids molecules, are key agents of energy conservation. These molecules are part of the endocannabinoid system (ECS) together with their receptors, the most well-known of which are the cannabinoid receptor type 1 (CB1) and 2 (CB2) as well as the transient receptor potential cation channel subfamily V member 1 (TRPV-1), and the enzymes in charge of their synthesis and elimination [20]. The ECS is involved in a plethora of physiological mechanisms comprising gametogenesis, implantation, and placentation [21]. Moreover, recent evidence suggests that an increase in CB1 levels is linked to some molecular and histological changes in CVs of preeclamptic placentae [22]. Bearing that in mind, it is likely that alterations in the ECS may be correlated with the morphological and structural changes triggered by other gestational pathologies such as GDM and SGA—a matter that has not been investigated so far.

In this scenario, the aim of this work was to better characterize the macromolecular and structural modifications occurring in the placenta of GDM and SGA patients, using a multidisciplinary approach, and to figure out if such changes are correlated with alterations in the endocannabinoid receptors.

## 2. Results

### 2.1. GDM and SGA Correlate with Increased Levels of Antioxidant Enzymes in Term Placenta

**Figure 1:**
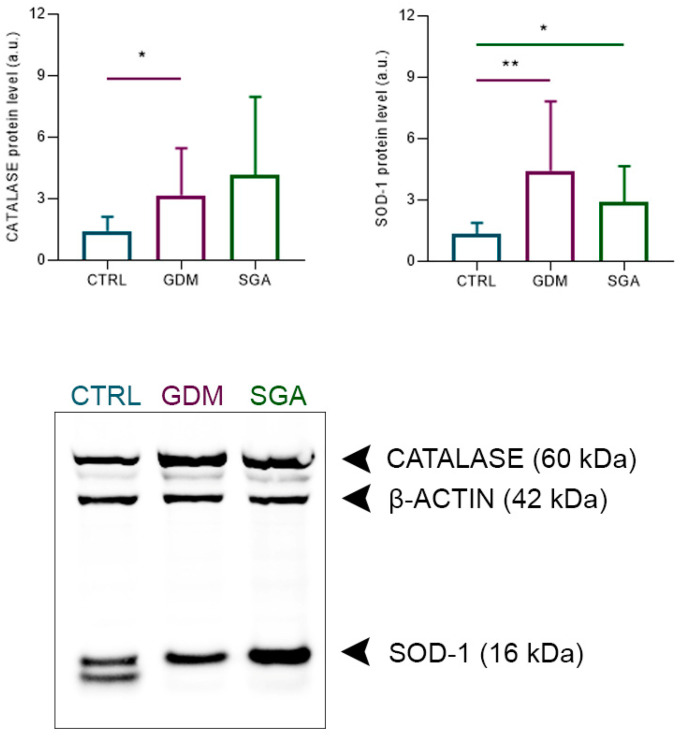
Relative levels of antioxidant enzymes in the control and pathological placenta slices. In the graphs, bars indicate the CATALASE (60 kDa)/β-actin (42 kDa) ratio and SOD-1 (16 kDa)/β-actin (42 kDa) ratio densitometric analysis ± SD of twelve independent replicates (*n* = 12). Asterisks indicate significant differences (* = *p* < 0.05; ** = *p* < 0.01) when comparing the pathologies to the control group. CTRL: control group (blue), GDM: gestational diabetes mellitus (purple), SGA: small-for-gestational-age (green).

The protein levels of the antioxidant enzymes CATALASE, which catalyzes the decomposition of H_2_O_2_ to H_2_O and O_2_, and SOD-1, which transforms the excess of O_2_^−^ in H_2_O_2_ and O_2_, were assessed in control and pathologic placenta samples (Figure 1). The results show that GDM increases the levels of both CATALASE and SOD-1 (*p* = 0.0328 and 0.0093, *n* = 12, respectively), whereas SGA triggers an increase in SOD-1 levels (*p* = 0.0157, *n* = 12).

### 2.2. Fourier Transform Infrared Imaging (FTIR) Analysis of CTRL, GDM, and SGA Placentae

**Figure 2:**
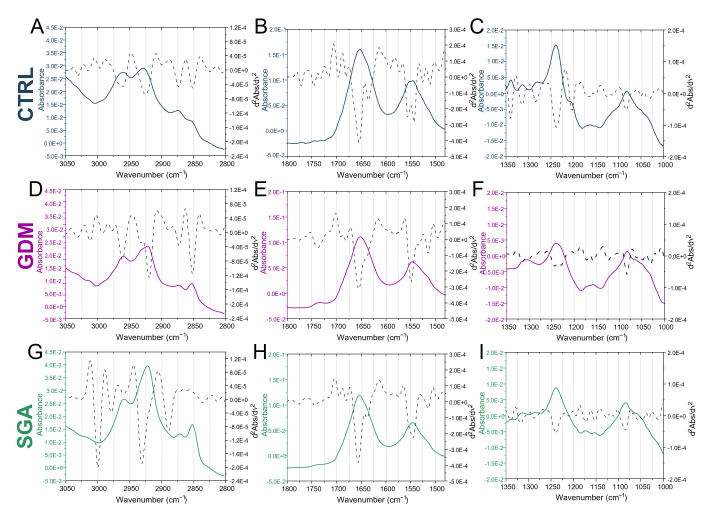
Vibrational analysis. Mean infrared spectra of control (CTRL, blue lines), gestational diabetes mellitus (GDM, purple lines), and small-for-gestational-age (SGA, green lines) samples. Spectra are displayed in absorbance (colored continuous lines) and second derivative modes (black dotted lines) in the 3050–2800 cm^−1^ (**A**,**D**,**G**), 1800–1480 cm^−1^ (**B**,**E**,**H**), and 1350–1000 cm^−1^ (**C**,**F**,**I**) intervals.

The mean infrared spectra of CTRL, GDM, and SGA experimental groups are shown in Figure 2 in the 3050–2800 cm^−1^, 1800–1500 cm^−1^, and 1330–1000 cm^−1^ spectral intervals, both in absorbance (respectively, continuous blue, purple, and green lines) and in second derivative (dotted black lines) modes. The most relevant IR bands were identified and assigned, according to the literature [23,24,25,26,27,28,29,30], as follows: 2960 cm^−1^ and 2870 cm^−1^ (asymmetric and symmetric stretching vibrations of CH_3_ moiety); 2925 cm^−1^ and 2850 cm^−1^ (asymmetric and symmetric stretching vibrations of CH_2_ moiety); 1740 cm^−1^ (stretching vibration of C=O ester moiety in triglycerides and phospholipids); 1660 cm^−1^ and 1635 cm^−1^ (Amide I band components of proteins, AI); 1555 cm^−1^ and 1540 cm^−1^ (Amide II band components of proteins, AII); 1320 cm^−1^ (α chain secondary structures of collagen); 1284 cm^−1^ and 1240 cm^−1^ (triple helix structures of collagen); 1204 cm^−1^ (amino acids lateral chains of collagen); 1172 cm^−1^ phosphodiester bond (COP groups); 1130 cm^−1^ and 1053 cm^−1^ (COH and C-O moieties of carbohydrates).

**Figure 3:**
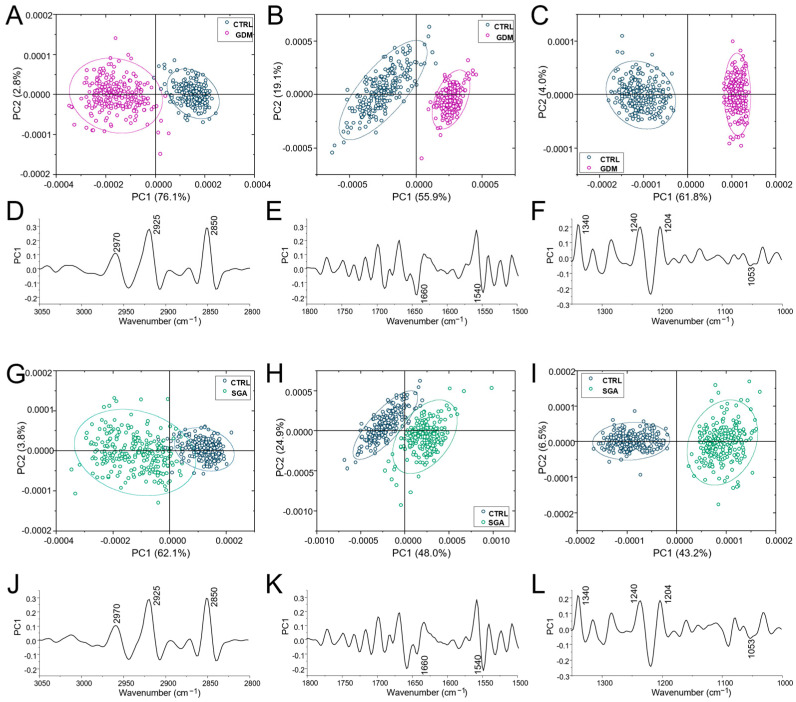
Multivariate analysis. PCA score plots and corresponding PC1 loadings calculated on CTRL, GDM, and SGA spectral populations in the 3050–2800 cm^−1^ (**A**,**D** and **G**,**J**), 1800–1480 cm^−1^ (**B**,**E** and **H**,**K**), and 1350–1000 cm^−1^ (**C**,**F** and **I**,**L**) intervals. For a better comprehension, PC1 loadings of the three selected intervals are displayed with different Y scales. CTRL: control group (blue), GDM: gestational diabetes mellitus (purple), SGA: small-for-gestational-age (green).

To have a deeper insight into the spectral changes triggered by GDM and SGA in the CVs, spectral data were then submitted to Principal Component Analysis (PCA) in the following ROI (Regions of Interest): 3050–2800 cm^−1^, 1800–1480 cm^−1^, and 1350–1000 cm^−1^ (Figure 3). The segregation between CTRL and GDM spectral data and CTRL and SGA ones was very evident in all the performed comparisons, especially those regarding the spectral range 1350–1000 cm^−1^ (Figure 3C,F,I,L).

### 2.3. SGA and GDM Alter Collagen Structures, Whereas Only SGA Decreases Collagen Deposition in Placental CV

**Figure 4:**
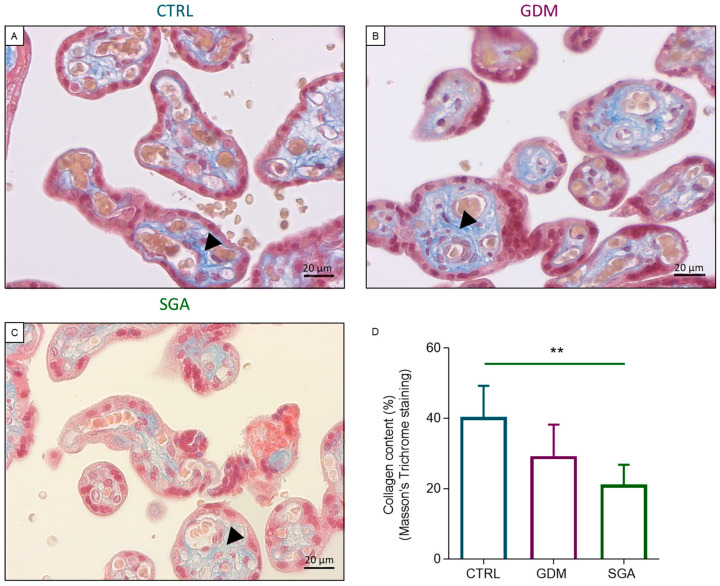
Collagen deposition analysis through histology in the three experimental groups. Masson’s Trichrome staining was performed to analyze the collagen deposition in the CVs (**A**–**C**). (**D**) Bars represent collagen deposition in the three conditions expressed as mean ± SD of five independent samples (*n* = 5). Asterisks indicate significant differences (** = *p* < 0.01) Representative histological images of the collagen deposition within the CV (black arrowhead) in CTRL (**B**), GDM (**C**), and SGA (**D**) placentae. CTRL: control group (blue), GDM: gestational diabetes mellitus (purple), SGA: small-for-gestational-age (green).

**Figure 5:**
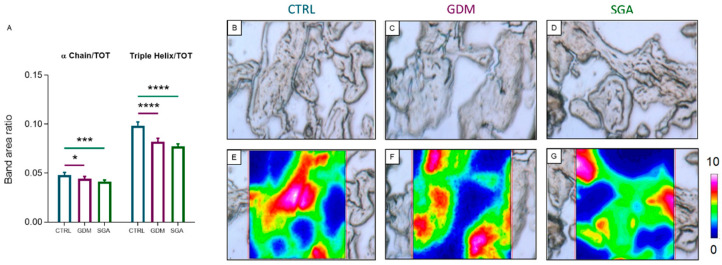
FTIRI analysis of collagen structure in CV sections of CTRL, GDM, and SGA experimental groups. (**A**) Statistical analysis of the following band area ratios: α Chain/TOT (relative amount of α chain secondary structures in collagen) and Triple Helix/TOT (relative amount of triple helix structures in collagen). TOT was the sum of the areas of all the peaks in the 1350–1000 cm^−1^ range. Values are reported as mean ± SD of five independent samples (*n* = 5). Asterisks indicate significant differences between CTRL and GDM and CTRL and SGA groups (* = *p* < 0.05, *** = *p* < 0.001, and **** = *p* < 0.0001). (**B**–**D**) Microphotographs of representative sections of CTRL, GDM, and SGA groups, and (**E**–**G**) corresponding false-color images showing the topographical distribution of collagen within the mapped areas; absorbances ranged from white/pink (representing the highest values) to blue/black (representing the lowest ones). CTRL: control group (blue), GDM: gestational diabetes mellitus (purple), SGA: small-for-gestational-age (green).

In order to investigate the collagen deposition in placental CVs of CTRL, GDM, and SGA, both Masson’s Trichrome staining (Figure 4A–D) and FTIRI analysis (Figure 5A–G) were performed. The histological analysis revealed a lower collagen deposition in SGA CVs compared to CTRL ones (*p* = 0.0047, *n* = 5), but not in the GDM CVs compared to those of CTRL group (*p* = 0.0858, *n* = 5). The assessment of collagen structure performed with FTIRI revealed a decrease in α chain (α Chain/TOT band area ratio) (*p* = 0.0331 and *p* = 0.0010, *n* = 5) and triple helix (Triple Helix/TOT band area ratio) (*p* < 0.0001 and *p* < 0.0001, *n* = 5) structures, in CVs from both GDM and SGA compared to CTRL ones.

### 2.4. GDM and SGA Modify the Lipidic Composition of Placental CVs

**Figure 6:**
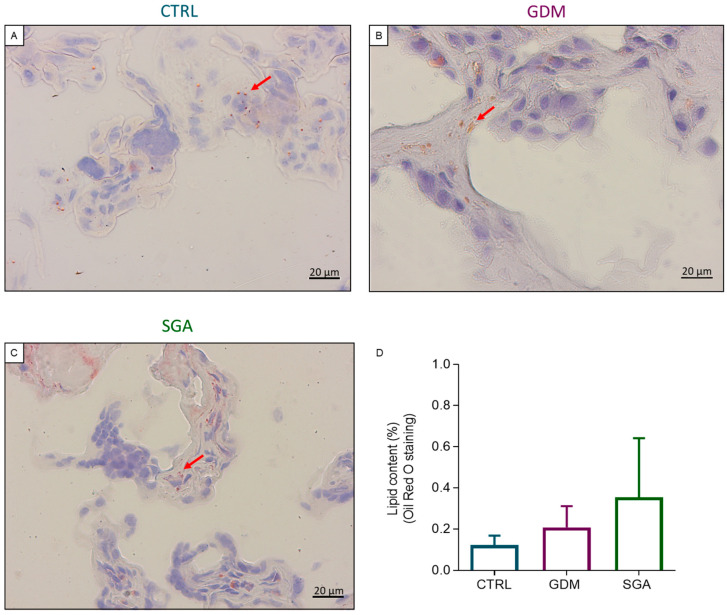
Lipid content analysis through histology in the three experimental groups. Representative histological images showing the lipid droplets (red arrows) within the CV sections (**A**–**C**). Analysis of lipid content in CV sections using Oil Red O staining (**D**). Bars represent lipid content in the three conditions expressed as mean ± SD of five independent samples (*n* = 5). CTRL: control group (blue), GDM: gestational diabetes mellitus (purple), SGA: small-for-gestational-age (green).

**Figure 7:**
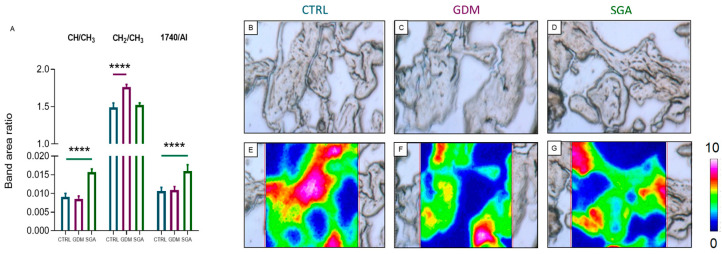
FTIRI analysis of the lipid content and peroxidation in CV sections of CTRL, GDM, and SGA experimental groups. (**A**) Statistical analysis of the following band area ratios: CH/CH_3_, unsaturation degree in lipid alkyl chains; CH_2_/CH_3_, length of lipid alkyl chains; 1740/AI, C=O ester moiety in lipid alkyl chains. Values are reported as mean ± SD of five independent samples (n = 5). Asterisks indicate significant differences between CTRL (blue) and GDM (purple) and CTRL and SGA (green) groups (**** = *p* < 0.0001). (**B**–**D**) Microphotographs of representative sections of CTRL, GDM, and SGA experimental groups, and (**E**–**G**) corresponding false-color images showing the topographical distribution of lipids within the mapped areas; absorbances ranged from white/pink (representing the highest values) and blue/black (representing the lowest ones).

The lipid composition of CTRL, GDM, and SGA placentae was evaluated by Oil Red O staining (Figure 6A–D) and FTIRI analysis (Figure 7A–G). The lipid content of placental CV did not vary among groups (*p* = 0.6692 and *p* = 0.1063, *n* = 5). The use of a more throughput methodology, the FTIRI, revealed an increase in lipid peroxidation (*p* < 0.0001, *n* = 5) and alkyl chain saturation (*p* < 0.0001, *n* = 5) only in SGA CVs, being the above-mentioned parameters in GDM CVs similar to those of CTRL group (*p* = 0.5298 and *p* = 0.4894, *n* = 5). On the contrary, the CVs of GDM placentae showed an increase in both length and saturation of the alkyl chains (*p* < 0.0001, *n* = 5) compared to CTRL. This change was not observed in SGA placental CVs (*p* = 0.3343, *n* = 5).

### 2.5. The Carbohydrate Content Is Increased in the CVs of GDM Placentae

**Figure 8:**
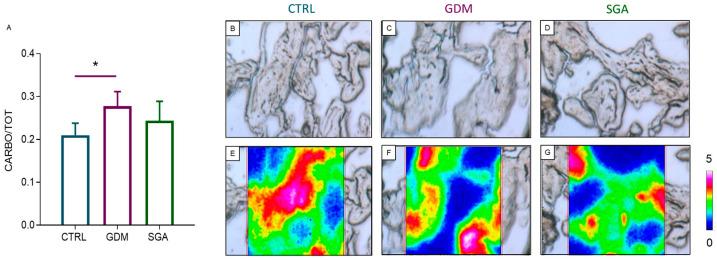
FTIRI analysis of the carbohydrate content in CV sections of CTRL, GDM, and SGA experimental groups. (**A**) Statistical analysis of the CARBO/TOT band area ratio, corresponding at the amount of total carbohydrates (TOT was the sum of the areas of all the bands in the 1350–1000 cm^−1^ range). Values are reported as mean ± SD of five independent samples (*n* = 5). Asterisks indicate significant differences between CTRL (control group, blue), GDM (gestational diabetes mellitus, purple), SGA (small-for-gestational-age, green) groups (* *p* < 0.05). (**B**–**D**) Microphotographs of representative sections of CTRL, GDM, and SGA groups, and (**E**–**G**) corresponding false-color images showing the topographical distribution of carbohydrates within the mapped areas; absorbances ranged from white/pink (representing the highest values) and blue/black (representing the lowest ones).

FTIRI analysis was performed to quantify the amount of carbohydrates in the placental CVs (Figure 8A–G): an increase was found only when comparing GDM to CTRL (*p* = 0.0221, *n* = 5), while no changes were found between SGA and CTRL (*p* = 0.2726, *n* = 5).

### 2.6. SGA Placental CVs Are Characterized by Lower Glycosylated Compounds and Higher Phosphodiester Bonds

**Figure 9:**
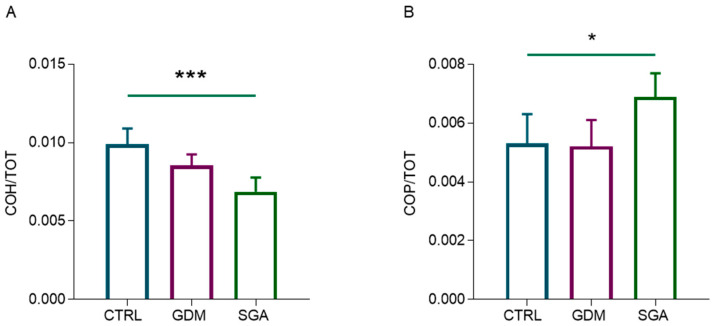
FTIRI analysis of glycosylated compounds and phosphodiester bonds in CV sections of CTRL, GDM, and SGA experimental groups. Statistical analysis of the following band area ratio: (**A**) COH/TOT (representing the relative number of glycosylated compounds) and (**B**) COP/TOT (representing the phosphodiester bonds). Results are reported as mean ± SD of five independent samples (*n* = 5), and asterisks indicate significant differences between CTRL and SGA groups (* = *p* < 0.05 and *** = *p* < 0.001). CTRL: control group (blue), GDM: gestational diabetes mellitus (purple), SGA: small-for-gestational-age (green).

The infrared spectroscopic analysis of SGA CVs revealed a decrease in glycosylated compounds (Figure 9A; *p* = 0,0003, *n* = 5) and a concomitant increase in phosphodiester bonds (Figure 9B; *p* = 0.0306, *n* = 5); conversely, no changes were observed comparing GDM and CTRL (*p* = 0.0554 and *p* = 0.9774, *n* = 5).

### 2.7. GDM and SGA Do Not Alter the Placental Levels of Endocannabinoid Receptors CB1 and TRPV-1

The study of two endocannabinoid receptors, CB1 and TRPV-1, in the whole placenta samples did not reveal any statistical differences between the levels of control and pathological samples (GDM *p* = 0.9346 and SGA *p* = 0.5094 for CB1; GDM *p* = 0.5313 and SGA *p* = 0.9029 for TRPV-1; *n* = 12) (Supplementary Figure S1A). Likewise, the immunohistochemistry analysis of CB1 levels (GDM *p* = 0.5225 and SGA *p* = 0.3883; *n* = 12) and TRPV-1 levels (GDM *p* = 0.5160 and SGA *p* = 0.8246; *n* = 12) in the CVs was not altered by either GDM or SGA (Supplementary Figure S1B).

## 3. Discussion

During pregnancy, the placenta fulfils a wide range of metabolic processes that mainly occur in the CV, a structure that represents the fetal–maternal interface [31]. For this reason, studying this portion of the placenta provides more accurate information about the alterations triggered by metabolic pathologies, such as GDM and SGA, than the bulk analysis of placentae [22]. In this regard, histology, immunohistochemistry, and FTIRI analyses can all be performed to study more specifically the changes at the CV level. The FTIRI is a throughput technique that allows the identification of alterations in the biochemical composition and/or conformation of the principal biomolecules in a tissue section without the use of any label, producing a fingerprint of the analyzed sample that can be useful to describe specific biomarkers of a disease [32]. Since the placenta is a heterogeneous organ, it presents some issues regarding the reliability and reproducibility of the data, so the sample collection should be performed from the same region, near the umbilical cord of the fetal side in our case, to reduce any methodological criticalities [33]. In addition, another strategy that can be applied is the enrollment of patients within a range of both age and gestational age, and the analysis of an equal number of placentae derived from Cesarean section and natural delivery, since it is already known that these two modes present changes regarding placenta structure, metabolism, gene expression, and protein levels [33]. In the present study, FTIRI and histology were performed on CVs from CTRL, GDM, and SGA term placentae to study the macromolecular alterations caused by these two pathologies. Moreover, to investigate the antioxidant enzymes and the potential involvement of the endocannabinoid system in these diseases, Western blot and immunohistochemistry were carried out in all groups.

Under physiological conditions, reactive oxygen species (ROS) are produced during the oxygen metabolism as by-products. However, it is noteworthy that high levels of ROS lead to oxidative distress. As a safeguard against this molecular damage, several non-enzymatic mechanisms interplay with antioxidant enzymes within the cells [34]. Still, if cells cannot cope with the oxidative stress, the derived physiological and molecular alterations can lead to the onset and progression of different pathologies [35]. Since ROS are produced by metabolic activity, a hyperglycemic environment, such as that present in GDM, has been associated with oxidative stress [35,36]. Even though the antioxidant response in the placenta has been evaluated, as reviewed by Turek et al., there is no consensus: some authors reported a decreased CAT activity in placentae of GDM patients who delivered through Cesarian section [37], whereas others revealed an increased *CAT* mRNA expression [18], but normal protein levels of CAT and SOD-1 were found in GDM placentae from Cesarean section compared with those from normal pregnancies after Cesarean section [38]. On the contrary, other studies showed an increased activity of SOD-1 [39] or both SOD-1 and CAT [40] in the placenta of GDM patients collected after Cesarean section. In the present study, higher levels of both CAT and SOD-1 were found in placentae of GDM, which are potentially linked to the increase in carbohydrates found by FTIRI in the GDM CVs of these patients. Taking into account that placentae were collected from a mixed population of Cesarian section and natural delivery, our data support, without the bias of the delivery type, that a hyperglycemic environment triggers the increase in the oxidative stress response. On the other hand, SGA is characterized by incompletely developed spiral arteries that cause ischemia-reperfusion, thus inducing oxidative stress [35]. Hence, fetal growth restriction was observed to increase the activity of SOD-1 in placentae [41] as well as the lipid peroxidation in both placenta and umbilical cord plasma by augmenting the level of malondialdehyde [42], which is an end-product of the fatty acid oxidation. This is in accordance with our findings that show higher levels of SOD-1 protein level in SGA bulk placentae and 1740/A1 ratio, which is a reliable biomarker for lipid peroxidation, in SGA CVs [43]. In addition, previous studies reported an increase in docosahexaenoic acid (DHA) levels in placentae from SGA pregnancy [44,45], a lipid that has been demonstrated in in vitro experiments to trigger lipid peroxidation and the subsequent oxidative stress induction [46]. Our FTIRI data also highlight the presence of a higher CH/CH3 ratio in SGA CVs that might well correlate with the increase in DHA previously reported. Despite the high levels of this lipid in the SGA placentae, the umbilical cord blood in SGA patients displays low levels of DHA [47]. This suggests that this lipid is not properly transported to the fetus, being accumulated in the placental environment, a matter that has been related to the decreased body weight of the SGA newborns [47]. Even if no changes in lipid content were found by the Oil Red O staining, the analysis of COH and COP in the SGA CVs revealed an impaired lipidic metabolism. High levels of COPs, and the concomitant low levels of COHs, likely indicate an increase in phosphorylated proteins, such as the phosphorylated serine/threonine kinase 11 (pSTK11) and phosphorylated AMP-activated protein kinase (pAMPK), which have already been described in placentae from SGA patients [48]. pSTK11 phosphorylates AMPK that, once active under energetically restricted conditions, induces the phosphorylation of other downstream proteins to switch the metabolic activity from the lipid biosynthesis to the β-oxidation [49]. In GDM, elevated levels of long chain fatty acids (LCFAs) detected by different metabolomic approaches have been suggested as potential biomarkers for the diagnosis [50]. LCFAs are essential for fetus growth [51] and their increase in GDM CVs possibly suggests an increase in their transport through the placentae, as already described and reviewed [52]. Moreover, a higher expression of *FATP2* mRNA in GDM syncytiotrophoblasts has been linked to an increase in long chain fatty acid acylation, transport, and trafficking [17]. The FTIRI assessment revealed an increase in CH2/CH3 ratio, indicating that in GDM the fatty acid chains are longer than in the CTRL group, supporting previous studies.

Considering the crucial role played by the ECS in the adipose tissue, the cannabinoid receptor 1 (CB1), activated by its ligands, regulates the lipoprotein lipase activity, leading to an increase in lipogenesis and adipogenesis [53]. Additionally, another receptor belonging to ECS, TRPV-1, has been reported to be activated by lipid peroxides during oxidative stress [54]. Bearing in mind that both GDM and SGA increase the lipid saturation and peroxidation, respectively, we have studied the levels of both CB1 and TRPV-1 not only in the bulk placentae but also in the CVs. However, neither the Western blot nor the immunohistochemistry revealed statistically significant differences in the receptor levels when comparing control and pathological samples. This is in contrast to recent results in which the CVs of preeclamptic placentae showed higher levels of CB1, but in this case, this alteration was more likely correlated with the fibrotic processes triggered by this pathological condition [22].

In the present study, the collagen deposition in the placenta was also assessed. A previous study performed on GDM patients found an increased fibrosis of villous stroma and interstitial collagen through Masson’s Trichrome staining [55], while our results, conducted on terminal CVs, and not on the bulk section, showed no changes in the collagen deposition comparing CTRL and GDM. A different scenario was present in SGA CVs, where Masson’s Trichrome staining evidenced a reduction in collagen deposition. Even though the levels of collagen have not been measured previously in the placenta from SGA patients, the expression of the genes codifying two proteoglycans (*BGN* and *DCN*), which are crucial for the collagen fibril and extracellular matrix assembly, was reduced in the first trimester of gestation in these patients [56]. Since this early change during gestation likely leads to the reduction in collagen in placentae, a more accurate assessment of the collagen structure has been performed for the first time in GDM and SGA placentae by FTIRI. This analysis showed that both pathologies trigger a reduction in the α-Chain, which stands for the collagen structural organization [30], being more evident in SGA than GDM, and of triple helix, a marker of collagen structure [30]. Therefore, even if only SGA led to a significant decrease in the collagen deposition, both pathologies induced changes in the collagen structure of placental CVs. An in vitro study reported that the induction of lipid peroxidation by ascorbic acid was related to an enhancement in collagen production [57]. Nevertheless, in our study by FTIRI, we have observed that SGA CVs showed higher levels of lipid peroxidation, but the collagen deposition decreases and the collagen secondary structure changes. Therefore, the correlation found in vitro by these authors in human fibroblasts cannot be applied to our in vivo study in human placentae.

The combination of the analysis performed revealed two specific phenotypes for each disease, confirming the accuracy of the FTIRI to identify some macromolecular biomarkers for these pathologies, pointing to the possible use of this technique as a diagnostic tool even at early stages of pregnancy. Furthermore, the possibility to perform this analysis in the CVs allowed the specific identification of macromolecular changes directly in the functional and metabolic unit of the placenta, avoiding the bias of the bulk placental assessments. Regarding the potential implication of the endocannabinoid system in the onset and/or progression of GDM and SGA, additional studies on other receptors are needed to shed light on this topic.

## 4. Materials and Methods

### 4.1. Ethics Declarations and Sample Collection

After delivery, term placenta samples were collected from informed and consent patients, at G. Salesi Hospital of Ancona (Italy), who have signed an appropriate consent form. This study was approved by Comitato Etico Regionale Marche ethical committee (n° CERM 241/2020).

Small placenta pieces were cut from the fetal side nearby the umbilical cord, under aseptic conditions, when afterbirth was concluded. For Western blot and FTIRI analyses, one portion of the placenta was stored at −80 °C, while for histology and immunohistochemistry, another portion was fixed in formalin solution (Bio Optica, Milan, Italy).

Diagnosis of GDM was performed using the oral glucose tolerance test (OGTT), while fetal length and weight were assessed for the diagnosis of SGA. Data used for the diagnosis are reported in Table 1.

### 4.2. Evaluation of Protein Levels by Western Blot

Proteins were extracted from frozen placenta samples sectioned from 12 CTRL, 12 GDM, and 12 SGA patients using Hanna’s buffer containing 0.125 M Tris-HCl pH 7.5, 4% (*w*/*v*) SDS, 20% (*v*/*v*) glycerol, and 10% (*v*/*v*) β-mercaptoethanol and supplemented with 1:10 Protease Inhibitor Cocktail (Sigma-Aldrich^®^, Milan, Italy). Both extraction and Western blot protocol were the same as described by Lombó and colleagues [22]. The membranes were incubated with the primary antibodies: Oxidative Stress Defense (ab179843, Abcam, Cambridge, UK), anti-β-actin (#4967, Cell Signaling TECHNOLOGY^®^, Danvers, MA, USA), anti-CB1 (ab259323, Abcam), and anti-TRPV-1 (ab3487, Abcam), and diluted 1:1000 in blocking solution overnight at 4 °C. Then, the Goat Anti-Rabbit IgG-HRP (Sigma-Aldrich^®^, Milan, Italy), diluted 1:2500 for 1 h at 30 °C, was used. Eventually, Image Lab Software (Bio-Rad, Milan, Italy) was used to digitalize the chemiluminescent signal and the densitometric analysis was carried out using the Fiji ImageJ Software 1.53t.

### 4.3. Immunohistochemistry and Image Analysis

Endocannabinoid receptors CB1 and TRPV-1 were detected in the CV sections of placentae samples (5 CTRL, 5 GDM, and 5 SGA), following the same immunohistochemistry protocol as previously described by Lombó and colleagues [22]. The sections were incubated overnight at 4 °C with a 1:100 dilution in blocking solution of the same antibodies (anti-CB1 and anti-TRPV-1) used for the Western blot, and then sections were incubated with a Goat Anti-Rabbit IgG H&L (Alexa Fluor^®^ 488; ab150077, Abcam) at 37 °C for 1 h, and nuclei were counterstained using DAPI-Aqueous Fluoroshield (ab104139, Abcam). A confocal microscope Nikon A1R was used to obtain the images of the CV, and the levels of CB1 and TRPV-1 were quantified using Fiji ImageJ Software 1.53t.

### 4.4. Histological Analysis of Collagen and Lipid Content

Masson’s Trichrome (Bio Optica, Milan, Italy) was performed following the manufacturer’s instructions in paraffin-embedded placentae (5 CTRL, 5 GDM, and 5 SGA) to assess the collagen content within the CVs.

Oil Red O staining was performed to evaluate the lipid content in frozen placenta samples (5 CTRL, 5 GDM, and 5 SGA), as previously described [22].

In both cases, the histological visualization was performed using an optical microscope Zeiss Axio Imager A.2 (Zeiss, Oberkochen, Germany) with an Axiocam 503 camera. The analysis was conducted from 5 images per sample using Fiji ImageJ Software 1.53t.

### 4.5. FTIRI Analysis

IR measurements were carried out by a Bruker INVENIO-R interferometer, coupled with a Hyperion 3000 Vis-IR microscope (15× condenser/objective) [22]. A Focal Plane Array (FPA) detector operating at liquid nitrogen temperature was used to acquire the IR images. Each IR image was 164 µm × 164 µm in size; it was composed of 4096 pixel/spectra, as a result of 256 scans; a spatial of 2.56 µm × 2.56 µm was obtained (Bruker Optics, Ettlingen, Germany).

From the same frozen samples used for Oil Red O staining, three thin sections (~10 µm thickness) were cut, deposited onto CaF_2_ optical windows (1-mm thick, 13-mm diameter), and let to air dry for 30 min. Each section was first visually inspected by a television camera to detect the areas of interest, on which to acquire the IR images in transmission mode in the 4000–800 cm^−1^ spectral range (spectral resolution 4 cm^−1^). Before starting each sample acquisition, the background spectrum was acquired on a clean portion of the CaF_2_ optical window with the same parameters described above. All IR images were pre-processed by the Atmospheric Compensation and Vector Normalization routines to avoid carbon dioxide and water vapor atmospheric contributions and to correct differences in section thickness (OPUS 8.1 software package, Bruker Optics, Ettlingen, Germany).

False-color images were generated by integrating pre-processed IR images under the 3000–2800 cm^−1^ (stretching vibrations of CH_2_ and CH_3_ groups in lipid alkyl chains); 1350–1200 cm^−1^ (vibrational modes of Amide III band, mainly attributed to collagen); 1140–1000 cm^−1^ (stretching vibrations of C-O groups in carbohydrates). A false-color scale was employed with white/light pink indicating zones with the highest absorbance values and black/dark blue indicating the zones with the lowest ones.

On each section, based on microphotograph’s evidence, a mapping subset of spectra representative of chorionic villi was extracted. Since the extracted spectra did not display scattering background and spectral artifacts, they were only submitted to two-points baseline linear fitting and vector normalization (OPUS 8.1 software, Bruker Optics, Ettlingen, Germany) [25].

The extracted spectra of CTRL, GDM, and SGA groups were also averaged and submitted to peak fitting procedure in the following Regions of Interest (ROI): 3050–2800 cm^−1^ (stretching vibrations of CH_2_ and CH_3_ groups in lipid alkyl chains); 1800–1500 cm^−1^ (Amide I and II bands of proteins, vibrational modes of peptide linkage); 1350–1000 cm^−1^ (vibrational modes of Amide III band, mainly attributed to collagen, and phosphate and carbohydrates groups). The number and position of the underlying bands were identified by second derivative minima analysis per each ROI and then fixed during fitting procedure using Gaussian functions (GRAMS/AI 9.1, Galactic Industries, Inc., Salem, New Hampshire). Finally, specific band area ratios were calculated using the integrated areas of some underlying bands of interest.

### 4.6. Statistical Analysis

Graph Pad Prism V8.0.1. (GraphPad Software, Inc., San Diego, CA, USA) was used to perform the statistical analyses. Once the normality of the data was assessed by the Shapiro–Wilk test, the following statistical analyses were performed: unpaired *t*-test with Welch’s correction for the parametric data and Mann–Whitney for non-parametric data. In all cases, the statistical significance was set at *p* < 0.05.

Concerning multivariate analysis of IR data, the processed spectra were converted in second derivative mode (Savitzky–Golay filter, 9 points of smoothing) and subjected to multivariate analysis, without any other further pre-processing. An unsupervised multivariate approach, Principal Components Analysis (PCA), was used to compare the spectral profiles of CTRL and GDM, and CTRL and SGA experimental groups (OriginPro 2018b software, OriginLab Corporation, Northampton, MA, USA) [23,25].

## Figures and Tables

**Table 1:** Clinical characteristics of the patients and parameters used for the diagnosis of GDM and Scheme 0. Was considered as statistically significant compared to CTRL group. * Corresponds to *p* < 0.05, ** corresponds to *p* < 0.01, and **** corresponds to *p* < 0.0001. pgi: post glucose intake.

	CTRL	GDM	SGA
Number of pregnant women	12	12	12
Parity			
Multiparous	9 (75%)	5 (41.66%)	6 (50%)
Primiparous	3 (25%)	7 (58.33%)	6 (50%)
Mode of delivery (%) Natural birth Cesarean section	6 (50%) 6 (50%)	7 (58.33%) 5 (41.66%)	5 (41.66%) 7 (58.33%)
Maternal age (years)	34 ± 3.51	34.58 ± 5.08	32.25 ± 5.06
Gestationl age (weeks)	39.41 ± 1.50	38 ± 1.83	38 ± 2.46
Fetal length (cm) * Fetal weight (g)	50.08 ± 3.80 * 3410.08 ± 405.33	47.75 ± 2.80 * 2945.41 ± 631.73	46.66 ± 3.72 * 2209.33 ± 616.01 ****
OGTT test Time 0 (Fasting) Time 1 (1 h pgi) * Time 2 (2 h pgi)	77.36 ± 9.58 115.54 ± 29.15 ** 102.36 ± 30.04	99.16 ± 20.36 ** 183.8 ± 33.43 **** 126.5 ± 45.10 **	81.75 ± 9.31 126.87 ± 36.82 ** 99.75 ± 35.45
Fetal sex			
Female	6 (50%)	8 (66.67%)	8 (66.67%)
Male	6 (50%)	4 (33.33%)	4 (33.33%)

## Data Availability

The data presented in this study are available on request from the corresponding author.

## Supplementary Materials

The following supporting information can be downloaded at: https://www.mdpi.com/article/10.3390/ijms24032240/s1.
