# Supplementary material for: Gestational Diabetes Mellitus and Small-for-Gestational-Age: An Insight into the Placental Molecular Biomarkers
Source: Int J Mol Sci. 2023 Jan 23;24(3):2240. doi: 10.3390/ijms24032240 (PMC9916826; doi:10.3390/ijms24032240)
Supplement: Supplementary file 1 [file ijms-24-02240-s001.zip › ijms-2166571-supplementary.pdf]

Gestational Diabetes Mellitus and Small-For-Gestational-Age: An Insight into the Placental Molecular Biomarkers

Christian Giommi [1,2,†], Marta Lombó [1,2,3,†], Nina Montik [4], Michela Paolucci [4], Valentina Notarstefano [1], Giovanni Delli Carpini [4], Andrea Ciavattini [4], Antonio Ragusa [5], Francesca Maradonna [1,2,*], Elisabetta Giorgini [1,‡], and Oliana Carnevali [1,2,*,‡]

1 Department of Life and Environmental Sciences, Università Politecnica delle Marche, 60131 Ancona, Italy
2 INBB—Consorzio Interuniversitario di Biosistemi e Biostrutture, 00136 Roma, Italy
3 Department of Molecular Biology, Faculty of Biology and Environmental Sciences, Universidad de León, 24071 León, Spain
4 Department of Odontostomatological and Specialized Clinical Sciences, Università Politecnica delle Marche, 60020 Ancona, Italy
5 Department of Obstetrics and Gynecology, Università Campus Bio Medico di Roma, 00128 Roma, Italy
† these authors contributed equally as first authors
‡ these authors contributed equally as co-last authors
* Correspondence: f.maradonna@staff.univpm.it (F.M.) and o.carnevali@staff.univpm.it (O.C.)

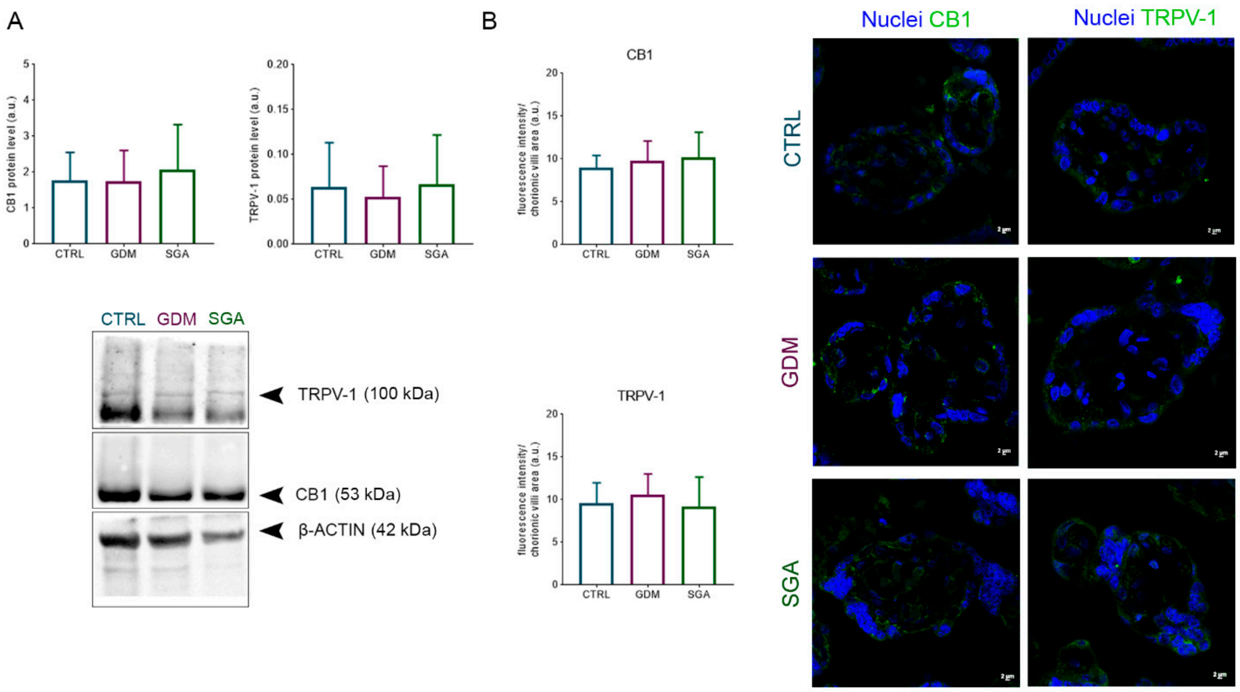

Supplementary Figure S1. Relative levels of endocannabinoid receptors in the control and pathological placenta slices (**A**). Bars represent the densitometric analysis of CB1 (53 kDa) / β-actin (42 kDa) ratio and TRPV-1 (100 kDa) / β-actin (42 kDa) ± SD of twelve independent replicates (*n* = 12). Analysis of the CB1 and TRPV-1 levels in chorionic villi by immunohistochemistry (**B**). Bars represent the fluorescence intensity per area of chorionic villi ± SD of five independent samples (*n* = 5). In confocal images, nuclei are marked with DAPI (blue) and CB1 or TRPV-1 are marked in green (Alexa Fluor® 488); scale bar = 2 µm.
